# Single-Dose Ibuprofen-Arginine as a Preventive for Pain, Edema, and Trismus After Impacted Lower Third Molar Surgery: A Randomized Split-Mouth Clinical Trial

**DOI:** 10.1055/s-0041-1735910

**Published:** 2021-12-22

**Authors:** Edith Umasi Ramos, Luan Pier Benetti, Júlio César Silva Oliveira, Ana Paula Farnezi Bassi

**Affiliations:** 1 Department of Surgery and Integrated Clinic, Division of Oral and Maxillofacial Surgery, São Paulo State University, Araçatuba Dental School, Araçatuba, São Paulo, Brazil

**Keywords:** arginine, ibuprofen, third molar, pain, edema

## Abstract

**Objective** We examined if the association of ibuprofen with arginine has a better anti-inflammatory effect on pain, edema, and trismus after surgery of the impacted mandibular third molar than ibuprofen alone.

**Materials and Methods** The study included 21 patients, 18 to 30 years of age, each with an impacted, and bilateral and symmetric third molar (total *n* = 21) that required transalveolar extraction. Patients were randomly assigned numbers from 1 to 21. Group A received ibuprofen-arginine as preoperative medication, while Group B received only ibuprofen. Both groups received the same postoperative medications: amoxicillin + acetaminophen. All patients were evaluated for pain at 6, 12, and 24 hours. They were evaluated for edema and trismus before surgery; immediately after surgery; and at 24, 48, and 72 hours postoperatively. Postoperative pain scores used the visual analog scale (BS-11). For facial edema and trismus, linear measurements used the method modified by Gabka and Matsumura.

**Statistical Analysis** For the evaluation of data between Group A and Group B, we used the statistical software SPSS version 22. The Shapiro-Wilk, analysis of variance, the Bonferroni comparisons, and the Wilcoxon test were used. All tests were based on a significance level of 0.05.

**Results** The study results reveal that the facial edema scores of Group A and Group B presented statistically significant differences ( *p* < 0.05), while for postoperative trismus, there was no statistically significant difference ( *p* > 0.05) between the scores of Group A and Group B.

**Conclusion** As a conclusion, we can state that the use of ibuprofen-arginine allows for significantly better control of pain and edema, and shows a tendency toward better recovery from trismus, although without statistical significance. Based on this, we can assert that arginine improves the anti-inflammatory power of ibuprofen, thus generating better tissue healing after surgery of the impacted third molar.

## Introduction

Extraction of the impacted lower third molar is a common dental procedure in oral and maxillofacial surgery, 1 and as a result of the damage to the surrounding tissues as well as the technique and degree of difficulty of the surgical procedure, 2 it causes postoperative sequelae such as pain, edema, and trismus 3 (also known as noninfectious complications 4 5 ). Surgeons worldwide use a variety of methods to minimize these sequelae, including medications. They may also use various therapies, including such recently studied techniques as kinesiology taping. 5 However, the control of postoperative complications remains an important research factor. 2 6 7 Among the most widely used preoperative medication protocols are nonsteroidal anti-inflammatory drugs (NSAIDs) and corticosteroids. When these are used, the need for prescribed postoperative analgesic is shown to be reduced, and there is also a reduction in postoperative complications. 2 7 8

Ibuprofen is one of the most widely used drugs in the NSAID category 9 ; it is taken at doses greater than 400 mg for control of moderate to severe pain. 10 Arginine is an essential amino acid that plays an important role in tissue healing, protein synthesis, and cellular immunity. 10 The pairing of ibuprofen with arginine has been used to yield greater therapeutic benefits than ibuprofen alone. 10 Various therapeutic approaches using ibuprofen-arginine have been found to provide faster control of pain compared with ibuprofen alone. 11 However, the effect of ibuprofen-arginine on other postoperative sequelae after third molar extraction (such as pain, edema, and trismus) has not been determined.

Ibuprofen (600 mg) with arginine is absorbed more quickly, since the maximum concentration is higher in a shorter time, 12 It is known that adequate preoperative dosage provides good pain control and reduced postoperative edema. 2 This amino acid represents the largest reserve of ATP energy levels and muscle regeneration, 13 14 useful in tissue healing and immunity. 13 14 15 Our hypothesis is that ibuprofen-arginine may allow for not only a lower intake of postoperative analgesics, but also less edema and trismus and therefore faster recovery from postoperative sequelae.

In this study, we first use ibuprofen-arginine (Spidufen, Zambom, São Paulo, Brazil) as a single dose 1 hour before surgery. The objective of this study is to evaluate the effect of ibuprofen-arginine, given before surgery of the impacted third molar, on postoperative pain, edema, and trismus, and ultimately to determine if arginine enhances the effect of ibuprofen on the inflammatory process after surgery.

## Materials and Methods

### Patient Selection

This study was a prospective, randomized, double-blind, controlled split-mouth trial to determine the effect of ibuprofen-arginine on pain, edema, and trismus after impacted third molar surgery in patients who attended the clinic and surgery facility at São Paulo State University (UNESP) School of Dentistry in Araçatuba, Brazil. This study was approved by the Brazilian unified database of research records involving humans for the entire CEP/CONEP system no. 24596919.7.0000.5420. All participants signed the informed consent prior to any procedure.

Inclusion criteria were (1) patients 18 to 30 years old in good health; (2) patients whose lower third molar positioning was bilateral and symmetric in accordance with the Pell & Gregory (Class 2B) and Winter (mesio-angulated) classification; 16 (3) patients whose impacted third molar apices did not touch the superior border of the inferior alveolar canal and whose risk for surgical extraction was therefore deemed low; and (4) patients whose impacted third molar apex extended beyond the superior border, but not the inferior border of the inferior alveolar canal and whose risk for surgical extraction was therefore deemed moderate 17 ( Fig. 2 ).

Exclusion criteria were (1) presence of any manifestation of inflammatory process, such as pericoronitis, cysts, and tumors, in the area of interest; (2) patients with a history of hypersensitivity to articaine and NSAIDs; (3) patients with any type of systemic disease; and (4) female patients in menstrual, gestational, or lactating periods. 18 19 20 A total of 65 volunteers were evaluated, of which 21 met the inclusion criteria ( Fig. 2 ). Each patient was randomly assigned a number from 1 to 21, for which Microsoft Excel and simple randomization procedures were used, divided into Group A (test group) and Group B (control group). 21

### Pharmacological Procedures

Preoperative medication was administered 1 hour before surgery. Neither the surgeon nor the patient knew at any time which prescription was being dispensed; all the drugs had the same appearance and were identified with codes. For Group A, the preoperative medication protocol was one sachet of 600 mg ibuprofen. Group B received one sachet of 1,155 mg ibuprofen-arginine, consisting of 600 mg ibuprofen + 555 mg arginine (Spidufen, Zambom, São Paulo, Brazil). The postoperative prescription for both groups was identical: one amoxicillin capsule (500 mg) every 8 hours for 7 days. Additionally, all patients were provided with a prescription for 750 mg paracetamol, a pain reliever. They were instructed to take the drug only if they had pain, and to maintain an interval of 6 hours between doses.

### Surgical Technique

The minimum time between each surgery was 21 days. 22 For intraoral antisepsis, 0.12% aqueous chlorhexidine digluconate solution was used, rinsing the mouth for 1 minute and. For extraoral antisepsis, 0.5% chlorhexidine alcohol solution was used. The anesthetic technique used was the lower alveolar, vestibular, and lingual nerve trunk block, with application of 4% articaine with 1:100,000 epinephrine. The ostectomy and odontosection were performed by means of a serrated truncated conical carbide cutter no. 702 and/or a spherical carbide drill no. 6 in high rotation; dental removal was completed with extractors. After this step, interrupted sutures were made with 4.0 nylon thread. For the postoperative period, the patients were instructed to consume a liquid, pasty, high-protein, and cold diet for the first 48 hours after surgery. All patients who developed postoperative complications, such as bleeding or dry or purulent alveolitis, were treated and excluded from the study. Postoperative evaluation was performed for aspects such as pain, swelling, and trismus.

### Pain Assessment

Pain was assessed at 6, 12, and 24 hours after surgery, using the visual analog scale (VAS), an 11-point scale (BS-11). 23 24 The study participants were instructed to self-assess their pain by marking the scale with an X. The scale consisted of 11 boxes ranging from 0 (no pain) to 10 (worst possible pain). The patients were instructed to ingest the first paracetamol tablet when they felt pain to measure the effect of the ibuprofen-arginine taken preoperatively and also to record the time and number of drugs ingested during the first 24 hours.

### Edema Assessment

A quantitative evaluation was used via linear measurement, following the method modified by Gabka and Matsumara. 25 These measurements were evaluated in the following periods: preoperative, immediate postoperative, and 24, 48, and 72 hours postoperatively since this last period is when the effect of inflammation generally reaches its peak. 26 The measurements were made by a calibrated examiner who was unaware of the type of medication used in the patients. The facial measurements were recorded in millimeters (mm). These were (1) from the tragus to the corner of the mouth; (2) tragus to pogonion; and (3) from the corner of the eye to the angle of the jaw.

### Trismus Assessment

Trismus was defined as the maximum opening of the mouth; it was evaluated by measuring the distance between the incisal edges of the upper and lower incisors with Vernier calipers. These measurements were recorded by a calibrated examiner in these periods: preoperative; immediate postoperative; and 24, 48, and 72 hours postoperatively. 25

### Sample Size Calculation

The sample size calculation was performed at www.lee.dante.br/pesquisa/amostragem/amostra , which indicated that a sample with nine patients per group would reach a power of 0.8. Thus, 21 patients were selected to participate of the study. Patients had both lower third molar teeth removed by the same surgeon with time interval of 21 days between both surgical procedures.

### Statistical Analysis

For the evaluation of data in our comparison between Group A and Group B, we used the statistical software SPSS version 22. The Shapiro-Wilk test was used to evaluate the distribution of the normality data, and the factor analysis of variance (ANOVA) for repeated comparisons. For the comparisons of the pain, edema, and trismus variables, the Bonferroni comparisons were used. For the number of analgesics and time of consumption of the first analgesic, the Wilcoxon test was used. All tests were based on a significance level of 0.05.

## Results

**Fig. 1:**
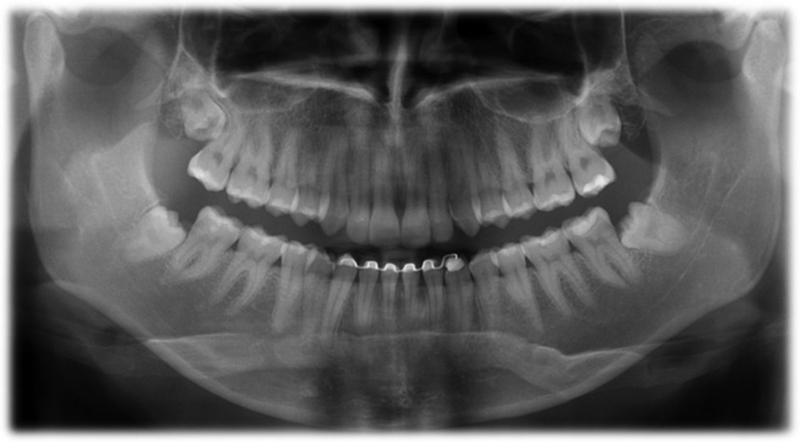
Panoramic radiography, third molar retained.

Of a total of 65 examinees, 21 participants met the study inclusion criteria. This group comprised 10 women and 11 men, ages 18 to 30 years, with a mean age of 25.4 ± 3.8 years ( Fig. 1 ). None of the patients had postoperative complications. The demographic characteristics, such as age, and surgery time, are presented in ( Table 1 ).

**Table 1:** Demographic characteristics and baseline

Variable	Group A ( *n* = 21)	Group B ( *n* = 21)	*p* Value a
Age (y) mean (SD)	25,1 ± 3.2	24.9 ± 3.5	0.13
Surgery time (min) mean (SD)	16.5 ± 4.1	16.2 ± 4.3	0.38

Abbreviations: *n* , number of patients; SD, Deviation standard.

a The *p* -value from Anova's test: significant difference ( *p* < 0.05).

The general means of pain, edema, trismus, and together with the means of the linear measures of edema, are presented in ( Table 2 ). With respect to pain, the global mean for Group A indicated a level of mild pain, and for Group B a moderate level; this difference was statistically significant ( *p* = 0.00). Regarding trismus, the decrease in mouth opening was from 15 to 18% in the two groups; however, the differences were not statistically significant ( *p* = 0.55). Finally, the global average of edema for Group A was significantly lower than Group B ( *p* = 0.00). It should be noted that the average of the linear measurements of edema of the tragus to the commissure were lower for group A; and of the angle of the jaw to the corner of the eye, were significantly lower for Group A (=0.00; Table 2 ).

**Table 2:** Summary of analgesic, edema, and trismus efficacy

Groups	Postoperative complication (mean + SD) *n* = 21					
	Pain	Trismus	Edema	Tg-Co	Tg-Pg	Co-M
Group A	1.8 ± 0.2	85 ± 1.8	2.9 ± 0.3	0.7 ± 0.1	4.0 ± 0.9	2.85 ± 0.4
Group B	3.8 ± 0.2	82 ± 2.9	5.1 ± 0.5	1.0 ± 0.3	6.6 ± 1.3	6.20 ± 0.7
p-value a	0.00 a	0.556	0.00 a	0.56	0.06	0.00 a

Abbreviations: Co-M, corner of the eye to the angle of the jaw; Group A, (Ibuprofen-arginine); Group B, (Ibuprofen); N, number patients; Tg-Co, tragus to the corner of the mouth; Tg-Pg, tragus to pogonion;

a Bonferroni's test: significant difference ( *p* < 0.05).

Analyzing the pain scale, it was observed that at 6 hours, the pain level was higher for both groups. However, for Group A the pain level was lower than for Group B, and the difference between the groups was significant ( *p* = 0.00; Table 3 ). In addition, the time period of ingestion of the first analgesic averaged 6.6 hours for Group A and 5.6 hours for Group B. However, these differences were not statistically significant ( *p* = 0.54; Table 4 ). Regarding the number of analgesics, there were significant differences between the groups ( *p* = 0.03), with a lower number of analgesics for Group A ( Table 4 ). Regarding the amount of articaine used in the patients, two tubes of 4% articaine with 1:100,000 epinephrine were used in 100% of the patients in both groups.

**Table 3:** Mean of analgesic and edema efficacy per times evaluated

Time	Pain sensation (mean ± SD)			Edema (sum) (percentual mean ± SD)			
	6 h	12 h	24 h	Immediately	24 h	48 h	72 h
Group A	2.6 ± 1	1.7 ± 1.2	1.1 ± 1.1	0.28 ± 0.1	2.68 ± 0.3	4.59 ± 0.5	4.29 ± 0.5
Group B	5.0 ± 1	3.9 ± 1.4	2.5 ± 2.5	1.55 ± 0.2	4.14 ± 0.5	7.36 ± 0.6	7.34 ± 0.8
*p* -Value a	0.00 a	0.00 a	0.015 a	0.00 a	0.031 a	0.00 a	0.00 a

Abbreviations: Co-M, corner of the eye to the angle of the jaw; Group A, (Ibuprofen-arginine); Group B, (Ibuprofen); *n* , number patients; Tg-Co, tragus to the corner of the mouth; Tg-Pg, tragus to pogonion.

a Bonferroni's test: significant difference ( *p* < 0.05).

**Table 4:** Mean of trismus, number of analgesics, and time of efficacy analgesic of single dose

Time	Trismus (sum) (percentual mean ± SD)				Analgesics (mean ± SD)	
	Immediately	24 h	48 h	72 h	Number of analgesics	First analgesic time
Group A	88.8 ± 13.5	78.6 ± 9.7	77.6 ± 10.3	81.2 ± 11.2	1.6 ± 0.96	400.4 ± 230.
Group B	84.4 ± 14.5	78.5 ± 10.6	74.6 ± 15.7	76.3 ± 14.1	2.6 ± 0.96	341.1 ± 162.
*p* -Value a	0.527	0.973	0.64	0.484	0.031 b	0.54

Abbreviations: Co-M, corner of the eye to the angle of the jaw; Group A, (Ibuprofen-arginine); Group B, (Ibuprofen); N, number patients; Tg-Co, tragus to the corner of the mouth; Tg-Pg, tragus to pogonion.

a Bonferroni's test for trismus: significant difference ( *p* < 0.05).

b Wilcoxon's test for number and time of analgesic: significant difference ( *p* < 0.05).

Regarding the evaluation of edema over time, it was observed that the mean edema in all periods was lower for Group A, with a significant difference in each of these periods: preoperative ( *p* = 0.00), 24 hours ( *p* = 0.03), 48 hours ( *p* = 0.00), and 72 hours ( *p* = 0.00; Table 3 ). Regarding trismus, it was observed that in all the time periods evaluated, there were no significant differences between both groups ( *p* = 0.55; Table 2 ); it was also observed that the least loss of mouth opening in the postoperative period was recorded in Group A (immediate, 88.8%; at 72 hours 81.2%). We can also highlight that the recovery of the mouth opening was proportionally higher in group A at 72 hours. However, there were no significant differences ( Table 4 ).

## Discussion

This study evaluated pain, edema, and trismus, postoperative complications that serve to evaluate the inflammatory process after the extraction of a retained third molar. 27 The control of postoperative morbidity continues to be an important factor in the recovery of patients. 6 Thus, the intensity of these complications varies depending on the surgical technique, degree of difficulty of the surgical procedure, and drug therapy. 27 Freudlsperger et al 28 reported that the higher the level of difficulty, the greater the complications; in the same way Zhoui et al 29 demonstrated that flap surgeries produce greater complications, regardless of the type of flap design. 30 In this study, impacted third molars with a moderate to severe degree of difficulty were selected. In addition, in the surgical technique, a triangular marginal flap was performed.

Another important factor is drug therapy. 27 A review by Chukwuneke and Onyejiaka 6 noted that surgeons worldwide use various pharmacological therapies, and that narcotic analgesics, NSAIDs, and corticosteroids are among the drugs used to treat pain and edema. 2 A review by Isiordia-Espinosa et al concluded that NSAIDs and dexamethasone are both effective for the management of postoperative complications. Furthermore, a systematic review by Torre et al 31 observed through qualitative and quantitative analysis that ibuprofen is more effective in relieving postoperative dental pain than other nonopioid analgesics.

However, with respect to the absorption time of ibuprofen, a review by Cattaneo et al 12 indicated that maximum plasma concentration is achieved 1.5 to 3 hours after ingestion. This represents a slow absorption rate, limiting the usefulness of ibuprofen for quick relief of acute pain. Likewise, a study by Werckweth et al 32 evaluated the role of cytochrome P450, which is responsible for the metabolism, absorption, and expression of NSAIDs. Using a dosage of 600 mg of ibuprofen, the study demonstrated that patients with poor metabolism had higher levels of pain. Because of such findings, pharmaceutical companies have developed different formulations to improve absorption 12 such as adding the amino acid L -arginine.

In a study by Dongseong et al, 33 it was shown that the ibuprofen-arginine combination is absorbed more quickly and has a higher maximum concentration than conventional ibuprofen. As a result, it takes effect sooner for the control of moderate-to-severe pain. 33 Furthermore, according to the review by Cattaneo et al, 12 the 600 mg concentration of Ibuprofen plus L -arginine was absorbed significantly faster than ibuprofen alone, in a maximum time of 18.6 versus 152.4 minutes, and with a maximum concentration of 71.8 and 43.9, respectively. But there is a lack of research that evaluates factors such as edema and trismus, which are part of the inflammatory process in third molar surgery.

According to the studies of Black et al 11 and Lau et al 34 that compared ibuprofen-arginine and ibuprofen in third molar surgery, ibuprofen-arginine was observed to facilitate faster pain control. Through this study, it was observed that ibuprofen-arginine, used as preoperative medication, had superior control over pain, and edema compared with ibuprofen alone, apparently enhancing the anti-inflammatory effect of ibuprofen. With respect to trismus, there was no significant difference. However, it was observed that with ibuprofen-arginine the mouth opening capacity was greater at 24 and 72 hours.

In general, the time range of maximum pain after third molar surgery is 6 to 8 hours. 23 In the study by Lau et al, 34 the first rescue medication of the group that used the 400 mg of ibuprofen-arginine preoperatively was at 7.3 hours. A single-dose randomized study by Desjardins et al 35 showed that the mean analgesic effect of 400 mg of ibuprofen arginate stayed high between the third and sixth hours. In this study, the first rescue medication was ingested at 5.6 and 6.6 hours after surgery, respectively, for the ibuprofen and ibuprofen arginate groups. The presumption is that arginine, in addition to improving the onset of action, allows a sustainable effect for a longer time.

Bjørnsson et al 36 conducted a clinical trial comparing 600 mg ibuprofen with 1,000 mg paracetamol, ingested four times a day for 3 days, starting 3 hours after the third molar was extracted. On the other hand, in the study by Lau et al, 34 the number of tablets ingested 24 hours after the surgical procedure, with 400 mg of ibuprofen-arginine used before and after surgery, were 2.53 and 2.17, respectively. In this study, postoperative medication was also evaluated in the first 24 hours; observing that for the ibuprofen-arginine group, it was significantly lower than for the ibuprofen-only group (1.6 and 2.6, respectively). Therefore, we can presume that the higher concentrations yield better control of postoperative pain, and lower doses can be used. In addition, it should be noted that for patients in the ibuprofen-arginine group, it was not necessary to ingest analgesics after the first 24 hours.

Because any pharmacological therapy can produce adverse effects, 32 there is more interest in nonpharmacological therapies. In the study by Jaron et al, 5 Kinesio Tape was used in the postoperative phase, demonstrating that it is an efficient method for reducing edema, trismus, and pain without generating adverse effects. With this study, we can also point out that arginine synthesizes nitric oxide, whose function is to protect the cells of the gastrointestinal tract and therefore reduce the adverse effects of ibuprofen on the gastric mucosa. 12

This randomized clinical trial took as a reference reliable method published in the literature for the evaluation of pain, edema, and trismus. Furthermore, this is an unpublished study due to the fact that the literature is mainly focused on evaluating pain; there is a lack of studies that evaluate edema and trismus. However, this study has limitations in relation to pain, first with the pain threshold, since this tolerance to pain seems to be influenced by genetic and psychosocial factors. In relation to edema and trismus, there is a limitation on the uniform management of postoperative care, since these could vary based on hygienic-dietetic measures and the physical measures followed by each patient. But these limitations do not invalidate the study, because it is difficult to achieve total control in this type of study.

## Conclusion

This study shows that arginine enhances the anti-inflammatory effect of ibuprofen. Therefore, 600 mg of ibuprofen-arginine is significantly superior compared with ibuprofen alone for control of pain and edema; it also allows better recovery of the ability to open the mouth from the second postoperative day. Regarding the effectiveness of the preoperative use of this drug, we observe that it has a sustainable effect over time, which allows the consumption of fewer postoperative analgesics. Despite these results, more studies are needed to determine the role of arginine in soft-tissue healing after oral surgery.

**Fig. 2:**
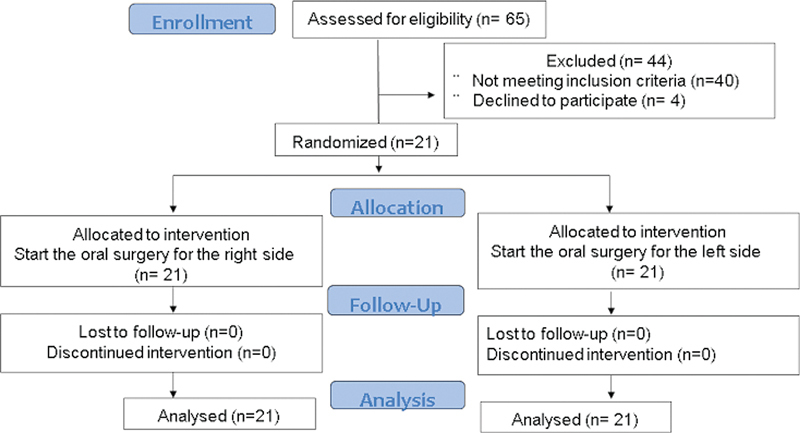
Consort Flow diagram.
